# Use of nitroglycerin for parallel transverse uterine cesarean section in patients with pernicious placenta previa and placenta accrete and predicted difficult airway: A case report and review of literature

**DOI:** 10.1097/MD.0000000000018943

**Published:** 2020-01-31

**Authors:** Yushan Ma, Yong You, Xiaoqin Jiang, Xuemei Lin

**Affiliations:** a Department of Anesthesiology, West China Second University Hospital, Sichuan University; b Key Laboratory of Birth Defects and Related Diseases of Women and Children (Sichuan University), Ministry of Education; c Department of Gynecology and Obstetrics, West China Second University Hospital, Sichuan University, Chengdu, Sichuan Province, China.

**Keywords:** nitroglycerin, parallel transverse uterine incision, pernicious placenta previa, postpartum hemorrhage, uterine relaxation

## Abstract

**Rationale:**

The incidence of obstetric hemorrhage due to pernicious placenta previa (PPP) and placenta accreta is currently increasing in China. Parallel transverse uterine incision (PTUI) cesarean section (CS) is a novel technique designed to avoid transecting the placenta and control postpartum hemorrhage during CS in these patients in our hospital. A key point of anesthesia management related to PTUI CS involves keeping the uterus relaxed. General anesthesia (GA) has often been performed, and inhaled volatile anesthetics have traditionally been recommended for this purpose; however, GA may be contraindicated in patients with difficult airways.

**Patient concerns:**

The patient was predicted to have a difficult airway, and GA may have resulted in potentially life-threatening complications. An alternative and safer method of achieving uterine relaxation during PTUI CS was thus required.

**Diagnoses:**

The patient was diagnosed with PPP, and a predicted difficult airway was suspected preoperatively.

**Interventions:**

PTUI CS was planned to control postpartum hemorrhage and preserve fertility during CS. Uterine relaxation during PTUI CS was achieved with intravenous nitroglycerin under combined spinal-epidural anesthesia.

**Outcome:**

Intravenous nitroglycerin and combined spinal-epidural anesthesia achieved uterine relaxation during the time from delivery of the neonate to making the second transverse incision in the lower segment of the uterus during PTUI CS. Both the parturient and neonate were well and were discharged 4 days later.

**Lessions:**

Intravenous nitroglycerin and combined spinal-epidural anesthesia may offer an alternative to GA for achieving uterine relaxation in patients with PPP and a predicted difficult airway undergoing PTUI CS to control postpartum hemorrhage.

## 1 Introduction

Pernicious placenta previa (PPP) and placenta accreta (PA) are the main causes of obstetric hemorrhage potentially resulting in maternal death.^[1]^ However, it is difficult for obstetricians to perform a traditional low transverse cesarean section (CS) in patients with PPP and PA^[2]^ because of the need to avoid transecting the placenta whilst removing it without direct visualization, which may result in catastrophic bleeding. Cesarean hysterectomy is the accepted procedure for uncontrolled intraoperative bleeding. A novel operative technique, parallel transverse uterine incision (PTUI) CS, was recently proposed in our hospital to avoid transecting the placenta while controlling obstetric bleeding and preserving the uterus.

The PTUI technique comprises 2 incisions and 2 temporary ligations of the uterus. A key point of anesthesia management related to PTUI CS involves keeping the uterus relaxed to allow continued placental perfusion and prevent placental separation during the time from delivery of the neonate to making the second transverse incision in the lower segment of the uterus. Inhaled volatile anesthetics, such as isoflurane or sevoflurane, have traditionally been recommended for uterine relaxation, and general anesthesia (GA) has thus often been applied for this purpose in obstetric emergencies. However, this procedure exposes obstetric patients with a potentially full stomach or difficult airway to unnecessary GA, with attendant risks and potentially life-threatening complications. An alternative and safer method of achieving uterine relaxation during PTUI CS technique may thus be required.

Nitroglycerin has demonstrated efficacy for inducing uterine relaxation to assist in resolving obstetric complications such as uterine inversion,^[3]^ manual extraction of the placenta,^[4]^ and ex utero intrapartum treatment (EXIT)^[5]^; however, its use in PTUI CS has not been reported. We speculate that intravenous (IV) nitroglycerin together with combined spinal-epidural anesthesia (CSEA) may be as effective as GA in patients undergoing PTUI CS.

We report our experience with a patient diagnosed with PPP and PA who underwent PTUI CS to control hemorrhage and preserve fertility. We also consider the use of IV nitroglycerin in conjunction with CSEA to relax the uterus during this procedure.

## 2 Case presentation

A healthy 39-year-old woman (gravida 4, para 1) visited our hospital as an outpatient because of PPP at 33–3/7 weeks of gestation. Her previous pregnancies involved 1 CS because of intrahepatic cholestasis of pregnancy in 2005, and 2 induced abortions in 2010 and 2012, respectively. Magnetic resonance imaging showed the placenta completely covering the internal cervical orifice. Based on these findings, the patient was considered to be at high risk for PPP. She subsequently underwent antenatal monitoring at 1-week intervals until 36 weeks of gestation, and then presented to the obstetric ward.

The surgical bleeding volume was expected to be large and the patient had a strong desire to maintain her fertility. A planned PTUI CS was; therefore, performed under CSEA at 36–2/7 weeks of gestation. She had a prior history of difficult airway and a predicted difficult airway was suspected by the anesthesiologist, and GA was therefore excluded. On arrival in the operating room, her blood pressure was 123/72 mmHg and her heart rate was 79 beats/min. CSEA was administered with 2.2 mL heavy bupivacaine (0.5%) into the subarachnoid space. The spinal needle was removed and the epidural catheter was inserted without complications. The patient was then placed in a supine position with left uterine displacement. Spinal block reached the level of T_7_ at 10 minutes.

A vertical abdominal incision was made, and the first transverse incision was then made near the uterine fundus and above the upper border of the placenta, without transecting the placenta. A healthy female infant weighing 2320 g, height 46 cm, was delivered smoothly from the first incision. The newborn's Apgar scores were 10 and 10 after 1 and 5 minutes, respectively. The placenta remained in the uterus avoiding iatrogenic partial separation, and a uterine fundal incision demonstrated minimal bleeding from the incision site, which was easily controlled by blood vessel ligation. Between delivery of the neonate and the second transverse uterine incision, 200 μg IV nitroglycerin was administered, followed by a continuous infusion of 0.3 μg/kg/min increased to 0.5 μg/kg/min until the obstetrician observed sufficient uterine relaxation. The patient's blood pressure was monitored continuously. The fundal uterine incision was sutured immediately. Shed blood from the surgical field was collected and washed using IV saline 0.9% to prepare for re-infusion. The patient's blood pressure remained at about 95–120/55–70 mm Hg throughout the 22-minutes suturing procedure, and her heart rate remained at 80 to 100 beats/min. One 6-mg bolus of ephedrine was administered for hypotension. Bilateral small openings were made in an avascular area of the ligament at the level of the cervix, and a narrow rubber tourniquet was passed through both openings and ligated tightly below the cervix to restrict uterine blood flow. The uterine body under the first fundus incision was also ligated tightly by another tourniquet to prevent bleeding from this incision. The nitroglycerin infusion was then stopped and a second transverse incision was made in the lower segment of the uterus, and the entire placenta was carefully removed under direct observation. The placenta was revealed to cover the entire anterior wall of the lower uterine segment. The placenta was completely removed and the tourniquet restricting blood flow to the uterine cervix and body was loosened, allowing confirmation of hemostasis. After confirming no obvious bleeding, the second incision in the lower segment of the uterus was closed by continuous sutures.

The total intraoperative blood loss was 1400 mL, and 421 mL of autologous red cells, washed with IV saline 0.9%, were re-infused back into the patient during the operation. A leukocyte filter was used to minimize the risk of amniotic fluid embolism during red cell re-infusion. The patient was transfused with crystalloid fluid (3000 mL) and hydroxyethyl starch (500 mL). Both the parturient and neonate recovered uneventfully and were discharged 4 days later.

## 3 Discussion

### 3.1 Main findings

CS is a common abdominal operation for the surgical delivery of a baby and the placenta.^[6]^ However, the increasing incidence of CS in China, particularly repeat CS with the liberalization of the 2-child policy, has led to a corresponding increase in the incidence of PPP with PA, which represents a major cause of maternal mortality. Recent clinical studies have focused on ways of preventing postpartum hemorrhage caused by PPP, and possible techniques vary depending on the clinical situation and surgeon preferences. Safe delivery is important for both the mother and infant. We report a patient with PPP and PA in whom the novel technique PTUI CS was used to control postpartum hemorrhage and preserve fertility.

In the current case, IV nitroglycerin in conjunction with CSEA improved the outcome by preventing placental separation and avoiding life-threating bleeding. The patient developed acceptable uterine relaxation between the delivery of the neonate and making the second transverse incision in the lower segment of the uterus. All the obstetric PTUI CS procedures were completed successfully, and the patient had no excessive blood loss based on clinical observation. IV nitroglycerin in conjunction with CSEA may thus be an effective alternative to GA for achieving uterine relaxation during PTUI CS

### 3.2 PTUI CS

The PTUI technique comprises 2 incisions and 2 temporary ligations of the uterus. The first transverse fundal incision was made near the uterine fundus and above the upper border of the placenta, without cutting through the placenta. The newborn was delivered safely and smoothly through this incision, which was then sutured rapidly. A rubber tourniquet was passed below the cervix and uterine body under the first fundus incision and ligated tightly. A second transverse uterine incision was then made in the lower segment of the uterus, followed by manual removal of the placenta under direct observation. Uterine relaxation was required from the time of delivery of the neonate to making the second transverse incision in the lower segment of the uterus. The new PTUI CS technique results in minimal bleeding from the first transverse fundal incision, while any bleeding that does occur can easily be controlled under direct observation.^[7,8]^

The total intraoperative blood loss in the current case was 1400 mL, which was only slightly more than that during routine transverse lower-segment CS in patients without PPP or PA. This case thus suggested that PTUI CS may reduce heavy bleeding in women with PPP and PA.

### 3.3 Purpose and method of uterine relaxation during the novel PTUI procedure

The key element to the success of PTUI CS involves maintaining uterine relaxation from the time of delivery of the neonate to making the second transverse incision in the lower segment of the uterus.^[7]^ In this situation, uterine relaxation is necessary to prevent placental separation from the endometrium and maintain placental perfusion.

Clinically, the inhaled volatile anesthetic, sevoflurane, was used to achieve uterine relaxation during PTUI CS in our hospital. High-dose (2–3 × minimum alveolar concentration [MAC]) inhaled volatile anesthetics have traditionally been recommended for this purpose. However, GA may not be appropriate for all obstetric emergencies, and is associated with risks of maternal morbidity and mortality. CSEA represents an alternative option that avoids complications, such as failed intubation and aspiration. Although not widely used, IV nitroglycerin in combination with CSEA has proven effective in patients needing rapid transient uterine relaxation to assist in obstetric complications.

The maintenance of uterine relaxation is key to successful PTUI CS. The present study provides the first reported use of IV nitroglycerin to manage PTUI during CS. Although uterine relaxation for PTUI CS was typically achieved with high-MAC sevoflurane in our hospital, it was also achieved successfully in this case using IV nitroglycerin in combination with CSEA. IV nitroglycerin plus CSEA may thus offer an alternative option for PTUI CS in patients in whom it is desirable to avoid GA, due to a difficult maternal airway, full stomach, or other reasons.

### 3.4 Dose of nitroglycerin for uterine relaxation in other obstetric emergencies

Uterine muscle relaxation is required to treat various intraoperative obstetric emergencies and has traditionally been achieved with potent inhalational anesthetics; however, nitroglycerin may be an attractive alternative for this purpose.^[9]^ The predictability, safety, and ease of IV administration of nitroglycerin have been firmly documented. It is a nitric oxide donor and an effective smooth muscle relaxant with a potent and short-lived tocolytic effect, and has been used at a variety of doses and via various routes. Its action following either sublingual^[4,10,11]^ or IV administration^[12,13]^ starts within 2 to 3 min, and mean peak nitroglycerin plasma concentrations are reached at approximately 6 to 7 min post-dose. Both administration routes thus demonstrate the same pharmacokinetic properties, though IV nitroglycerin is the preferred route for uterine relaxation in obstetric emergencies.

Its rapid onset, short half-life, and minimal side effects mean that a wide range of nitroglycerin doses have been proposed for uterine relaxation.^[5,14]^ The different doses used in different studies could explain the different effects. In the current Case 1, the obstetrician noted good conditions for PTUI during CSEA after an IV nitroglycerin bolus of 200 μg followed by infusion of 0.3 to 0.5 μg/kg/min. Axemo et al also reported effective uterine relaxation after a bolus injection of 100 to 200 μg nitroglycerin,^[15]^ while Kashanian et al found that 200 μg of IV nitroglycerin allowed effective placental delivery with no serious adverse effects.^[12]^ However, another study by Visalyaputra et al reported that the same amount of nitroglycerin was ineffective in facilitating the delivery of a retained placenta,^[16]^ with success rates of 15% in the study group and 20% in the control group. If uterine relaxation is inadequate, it may be necessary to increase the infusion rate of nitroglycerin or use additional boluses. However, the most widely recommended schedule includes a loading bolus of 100 to 200 μg, repeated 2 or 3 times if necessary, followed by an infusion of 1 to 20 μg/kg/min as needed, to maintain relaxation.

However, nitroglycerin has some possible side effects, including hypotension and tachycardia. Hemodynamically, nitroglycerin has a mild but significant effect on both pulse rate and blood pressure. Continuous low-dose nitroglycerin infusions (0.1–0.2 μg/kg/min) generally do not cause hypotension, though the dosages used (50–100ug) were lower than the 500 μg bolus used by Peng et al^[17]^ for manual removal of the placenta. However, boluses >500 μg may be associated with an increased risk of significant hypotension, suggesting that IV nitroglycerin may depress the circulation and should not be given to patients with uncorrected hypovolemia. Continuous hemodynamic monitoring is required and ephedrine should be administered if hypotension occurs. Headache is another complication of nitroglycerin, though the risk is minimal at low dosages. Nitroglycerin administration did not cause headache in the current patients but did result in a slight drop in blood pressure and a related increase in heart rate.^[9]^ Finally, Saroa et al observed unexpected hypoxemia following bolus nitroglycerin administration, and it should thus be used with caution in individuals susceptible to hypoxemia.^[18]^ However, although nitroglycerin is associated with dose-dependent side effects, these are likely to be short-term and self-limiting because of the short half-life of IV nitroglycerin.

### 3.5 Indications for nitroglycerin in intraoperative obstetric emergencies 

A safe and reliable method of achieving transient uterine relaxation during intraoperative obstetric emergencies is needed. Nitroglycerin has been reported to be safe and effective for obtaining good uterine relaxation; however, the indications for nitroglycerin use need to be emphasized to ensure its safe administration.

(1) Use of nitroglycerin to manage PTUI CS. PTUI CS is a novel technique to control bleeding and preserve the uterus in patients with PPP. The present study provides the first reported use of IV nitroglycerin to manage PTUI during CS.
(2) Use of nitroglycerin to deliver a retained placenta. Retained placenta affects 0.5% to 3% of women following delivery, with considerable morbidity if left untreated. Nitroglycerin can be used to relax the uterine smooth muscles as a last resort in patients with a retained placenta,^[10]^ and may minimize the need for manual removal of the placenta under anesthesia.^[9,19]^ Nitroglycerin has been used in obstetric emergencies for over 100 years, and was reported to achieve uterine relaxation for removal of a retained placenta by Peng et al in 1989.^[17]^ Sublingual or IV injection of nitroglycerin reduced the need for manual removal of the placenta and reduced bleeding compared with placebo following failure of oxytocin in patients in the third stage of labor,^[20]^ and all patients had rapid effective uterine relaxation after IV injection of 100 to 200 μg nitroglycerin.
(3) Use of nitroglycerin for EXIT. The aim of the EXIT procedure is to manage life-threatening neonatal emergencies and thereby increase the survival rate at delivery.^[13]^ As long as uterine relaxation and uteroplacental circulation are maintained until the fetal airway is secured, anesthesia can be performed in various ways. To date, GA has been advocated for management of the EXIT procedure; however rare maternal conditions may necessitate changes to this routine anesthesia care. Benonis et al^[5]^ reported the use of continuous spinal anesthesia in conjunction with IV nitroglycerin for EXIT in a woman with congenital arthrogryposis multiplex. An infusion of nitroglycerin (16 μg/kg/min) was also initiated to provide uterine relaxation in a patient with a family history of malignant hyperthermia.^[21]^
(4) Use of nitroglycerin for uterine inversion. Uterine inversion is a rare complication following vaginal delivery. However, nitroglycerin may also be used to treat this obstetric emergency. Altabef et al reported the use of nitroglycerin to allow replacement of a completely prolapsed, inverted uterus after normal vaginal delivery.^[22]^ Bayhi et al^[23]^ reported a case of uterine inversion occurring during CS with spinal analgesia, treated with IV nitroglycerin without GA. The uterus was unintentionally inverted during manual extraction of the placenta, but IV nitroglycerin (200 μg) was injected and the uterus relaxed substantially within 1 minute, allowing successful uterine reversion.

## 4 Conclusion

We report a patient with PPP and PA and predicted difficult airway in whom PTUI CS was used to control postpartum hemorrhage and preserve fertility during CS. PTUI CS was completed successfully without excessive blood loss. IV nitroglycerin and CSEA achieved uterine relaxation during the time from delivery of the neonate to making the second transverse incision in the lower segment of the uterus. In high-risk patients with GA, we strongly recommend IV nitroglycerin in combination with CSEA as an alternative effective method for achieving uterine relaxation during PTUI CS.

## Acknowledgment

The authors would like to thank Lan Wu, for their help in the work.

## Author contributions

**Data curation:** Xiaoqin Jiang.

**Investigation:** Yong You, Xuemei Lin.

**Methodology:** Xuemei Lin.

**Project administration:** Yong You.

**Resources:** Yushan Ma.

**Supervision:** Xuemei Lin.

**Writing – original draft:** Yushan Ma.

**Writing – review & editing:** Xiaoqin Jiang.
